# Infant outcome after active management of early‐onset fetal growth restriction with absent or reversed umbilical artery blood flow

**DOI:** 10.1002/uog.23101

**Published:** 2021-06-02

**Authors:** E. Morsing, J. Brodszki, A. Thuring, K. Maršál

**Affiliations:** ^1^ Pediatrics, Department of Clinical Sciences Lund University Lund Sweden; ^2^ Obstetrics and Gynecology, Department of Clinical Sciences Lund University Lund Sweden

**Keywords:** ARED flow, fetal growth restriction, long‐term follow‐up, neurodevelopmental impairment, survival, very preterm birth

## Abstract

**Objective:**

To describe the short‐ and long‐term outcomes of infants with early‐onset fetal growth restriction (FGR) and umbilical artery absent or reversed end‐diastolic flow (AREDF), delivered before 30 weeks' gestation and managed proactively.

**Methods:**

This was a retrospective cohort study of fetuses delivered for fetal indication before 30 completed weeks' gestation that had early‐onset FGR (defined as estimated fetal weight more than 2 SD below the mean) with AREDF in the umbilical artery (FGR group), at the level‐3 perinatal unit in Lund, Sweden, between 1998 and 2015. Perinatal outcome and neurodevelopment at ≥ 2 years of age in surviving infants were compared with those of a group of infants without small‐for‐gestational‐age birth weight or any known fetal Doppler changes delivered before 30 weeks in Lund during the corresponding time period (non‐FGR group). In the FGR group, the main indication for delivery was the Doppler finding of AREDF in the umbilical artery.

**Results:**

There were 139 fetuses (of which 26% were a twin/triplet) in the FGR group and 946 fetuses (of which 28% were a twin/triplet) in the non‐FGR group. The FGR infants had a median birth weight of 630 g (range, 340–1165 g) and gestational age at birth of 187 days (range, 164–209 days), as compared with 950 g (range, 470–2194 g) and 185 days (range, 154–209 days), respectively, in the non‐FGR group. The rate of fetal mortality did not differ between the two groups (5.0% and 5.4% in the FGR and non‐FGR groups, respectively). All seven intrauterine deaths in the FGR group occurred before 26 weeks' gestation. In the FGR group compared with the non‐FGR group, severe intraventricular hemorrhage was less frequent and bronchopulmonary dysplasia and septicemia were more frequent (*P* = 0.008, *P* < 0.001 and *P* = 0.017, respectively). In the FGR group, the survival rate at 2 years (83% of liveborn infants) and the rate of cerebral palsy (7%) did not differ significantly from those in the non‐FGR group (82% and 8%, respectively). The rate of survival without neurodevelopmental impairment was higher in the non‐FGR group (83%) than in the FGR group (62%) (*P* < 0.001), as well as in infants in the FGR group delivered at or after 26 weeks (72%) compared with those delivered before 26 weeks (40%) (*P* = 0.003). Within the FGR group, outcomes were similar between twins and singletons and, in those who survived beyond 2 years, outcomes were similar between fetuses with absent and those with reversed end‐diastolic flow in the umbilical artery.

**Conclusions:**

Infants delivered very preterm after severe FGR with AREDF in the umbilical artery had a similar rate of survival as did non‐FGR infants of corresponding gestational age; however, they were at higher risk of neurodevelopmental impairment, the risk being most pronounced following birth before 26 weeks. Gestational age remains an important factor associated with the prognosis of early‐onset FGR; nevertheless, the present results support the hypothesis, which should be tested prospectively, that fetuses with early‐onset FGR and umbilical artery AREDF may benefit from early intervention rather than expectant management, and that umbilical artery Doppler findings could be incorporated into clinical protocols for cases very early in gestation. © 2020 The Authors. Ultrasound in Obstetrics & Gynecology published by John Wiley & Sons Ltd on behalf of International Society of Ultrasound in Obstetrics and Gynecology.

**CONTRIBUTION**

**What are the novel findings of this work?**

Our findings support the hypothesis that proactive delivery of very preterm fetuses with fetal growth restriction (FGR) before 30 weeks' gestation based on a finding of absent or reversed end‐diastolic flow in the umbilical artery might prevent intrauterine demise. The long‐term neurodevelopmental outcome of these infants was comparable with, or better than, that reported in other studies with more conservative perinatal management.

**What are the clinical implications of this work?**

After verification of the present findings in prospective studies, proactive perinatal clinical protocols, taking into account umbilical artery Doppler when deciding on the timing of delivery before 30 weeks, might improve the outcome in very severe early‐onset FGR.

## INTRODUCTION

Early‐onset fetal growth restriction (FGR) is most often diagnosed based on a finding of a small‐for‐gestational‐age (SGA) fetus with abnormal blood flow in the umbilical artery recorded by Doppler ultrasound. FGR is associated with an increased risk of intrauterine death and with suboptimal neurodevelopment in survivors^1^, ^2^. In growth‐restricted infants delivered very preterm, the negative effects of FGR on development after birth are combined with those of prematurity. Gestational age (GA) at delivery is considered to be the strongest predictor of postnatal development^3^. However, in the clinical context, attempts to prolong early‐onset FGR pregnancies have to be balanced against the risk of intrauterine demise.

The finding of absent or reversed end‐diastolic flow (AREDF) in the umbilical artery in FGR is recognized as a sign of severely impaired placental perfusion, and is an indicator of adverse outcome^4^, ^5^. Nevertheless, in early‐onset FGR, up to 30–32 weeks' gestation, umbilical artery Doppler is usually not part of management protocols, and the clinician relies on other parameters of fetal condition, such as Doppler velocimetry of the ductus venosus (DV), fetal heart rate (FHR) tracing or biophysical profile^6^. In contrast, since 1998, at the level‐3 Perinatal Center at the Skåne University Hospital in Lund, we have adopted a proactive approach and used a clinical management protocol for early‐onset FGR in which umbilical artery Doppler is the most important parameter for determining the timing of delivery.

Early‐onset FGR with AREDF is a rare condition, and most of the published studies therefore comprise small groups of patients, not achieving adequate statistical power. In the present retrospective study, we analyzed the perinatal and long‐term neurodevelopmental outcomes of FGR cases with AREDF that were delivered before 30 weeks. Previously, we reported our experience from the first 6 years of proactive management^7^ and, subsequently, published the results from structured follow‐up examinations of a subgroup of 34 infants, in comparison with two matched control groups^8^, ^9^, ^10^, ^11^. The present larger study allows additional analysis over time of the outcome of twin *vs* singleton fetuses, fetuses delivered before *vs* those delivered after 26 weeks' gestation and fetuses with absent end‐diastolic flow (AEDF) *vs* those with reversed end‐diastolic flow (REDF).

## PATIENTS AND METHODS

We conducted a retrospective cohort study of fetuses delivered for fetal indication before 30 completed weeks' gestation that had early‐onset FGR with AREDF in the umbilical artery (FGR group) at the Department of Obstetrics and Gynecology in Lund, Skåne University Hospital, between 1998 and 2015. The patients were included in the study at the first occurrence of AREDF. Perinatal outcomes and neurodevelopment in surviving infants were compared with those of a group of infants without SGA birth weight or any known fetal Doppler changes, delivered before 30 weeks in Lund during the corresponding time period (non‐FGR group).

The Perinatal Center in Lund is a referral center with about 4000 deliveries per year, serving a background population of 1.8 million inhabitants in the Southern Swedish Health Care Region. Since 1998, a proactive clinical protocol has been used for the management of very preterm fetuses with FGR, aiming to avoid severe fetal hypoxia and deterioration in fetal condition. The protocol indicates delivery at the time of occurrence of REDF in the umbilical artery, and/or if there are rapidly progressing changes in the DV blood velocity waveform and/or pathological changes in FHR (loss of variability, late decelerations). After 26 weeks, AEDF in the umbilical artery is an indication for delivery following a full course of antenatal steroid treatment. One of the main principles is to deliver before the pathological FHR or DV Doppler changes occur.

### Perinatal data

Pregnant women with early‐onset FGR were hospitalized for intensive fetal surveillance and for antenatal steroid treatment (12 mg betamethasone on two consecutive days). FGR was defined as ultrasound estimated fetal weight more than 2 SD below the mean of the Swedish reference curve for intrauterine growth^12^, ^13^ and AREDF in the umbilical artery. All pregnancies were dated by ultrasound during the first half of gestation, most of them by routine fetometry at 17–18 postmenstrual weeks. Doppler flow velocity signals from the umbilical artery, fetal middle cerebral artery (MCA), DV, umbilical vein and both maternal uterine arteries were recorded using a Philips HDI 5000 or Philips IU22 ultrasound system (Philips Medical Systems, Bothell, WA, USA). Abnormal DV flow was defined as absent or reversed flow during the a‐wave. Fetal brain sparing was diagnosed as MCA pulsatility index more than 2 SD below the mean of the reference^14^. Uterine artery Doppler recordings were evaluated using the uterine artery score^15^, with scores of 3 or 4 being considered abnormal. In all cases, the Doppler recordings were performed in a standardized manner by experienced sonographers at the Laboratory for Obstetric Doppler Velocimetry. FHR recordings and Doppler velocimetry were performed daily or every second day after the diagnosis of AREDF. Fetuses with an antenatally diagnosed chromosomal abnormality or severe malformation and fetuses with twin‐to‐twin transfusion syndrome were excluded from the study. One fetus with AEDF diagnosed at 29 + 5 weeks and delivered at 30 + 0 weeks was not included.

The data on mothers, pregnancies, deliveries and neonates were retrieved from patient records, and the results of Doppler examinations were retrieved from the clinical database at the Laboratory for Obstetric Doppler Velocimetry. Live birth was defined according to the World Health Organization as a birth with any sign of life^16^. SGA was defined as birth weight more than 2 SD below the mean of the Swedish standard^13^.

### Neonatal data

Neonatal active care included resuscitation, early surfactant treatment, ventilator treatment and early enteral feeding with human milk. Treatment for low arterial blood pressure was given as either volume expansion or inotropic drugs. All cases of respiratory distress syndrome (RDS) were X‐ray verified. Bronchopulmonary dysplasia (BPD) was defined as requirement for supplemental oxygen at a postmenstrual age of 36 weeks. Septicemia was defined as the presence of bacteria in blood culture and elevated level of C‐reactive protein (reference value < 5 mg/L), and late‐onset septicemia was defined as clinical symptoms occurring after 6 days of age. Necrotizing enterocolitis (NEC) was defined as modified Bell's criteria Stage ≥ IIB^17^, and severe intraventricular hemorrhage (IVH) was determined by cranial ultrasound (intraventricular blood > 2/3 of the lateral ventricle in a sagittal section) and/or periventricular hemorrhagic infarction^18^. Cranial ultrasound was performed by a trained neonatologist or radiologist on postnatal days 1, 3 and 7, on postnatal week 3 and at term. Severe retinopathy of prematurity (ROP) was defined as ROP Stage ≥ 3^19^.

### Follow‐up data

Cerebral palsy (CP) was diagnosed at or after 2 years of age and categorized according to the Gross Motor Function Classification System (GMFCS)^20^. Information on cognitive function was obtained from the formal developmental assessment at 2 years of corrected age (Bayley Scales of Infant and Toddler Development, 2^nd^ or 3^rd^ edition (Bayley II or III))^21^, from cognitive evaluation using the Wechsler Preschool and Primary Scale of Intelligence (WPPSI‐III)^22^ or the Wechsler Intelligence Scale for Children (WISC)^23^, and from clinical assessment by a neurologist/neonatologist as recorded in the children's charts.

Records from the Pediatric Habilitation Service, which is a referral institution for children with CP, autistic disorder or moderate to severe cognitive deficits, were scrutinized. The diagnosis of autistic disorder was based on a record of ICD‐10‐CM code F84.0 in the pediatric charts. Attention deficit disorder was defined as a child being treated with methylphenidate. Hearing impairment was defined as unilateral or bilateral sensorineural hearing impairment.

Neurodevelopmental impairment (NDI) was defined as any of the following: CP (GMFCS level > 2), cognitive delay (Bayley II score < 70, Bayley III score < 85, WPPSI‐III or WISC IV score < 70 or developmental delay/presence of cognitive impairment documented in the Pediatric Habilitation Service records), severe hearing impairment (dependence on hearing aids or worse) and blindness. Body weight at 2 years of age more than 2 SD below the mean of the Swedish growth charts was considered as growth failure^24^.

### Statistical analysis

Statistical analysis was performed using statistical software packages SPSS 25.0 (SPSS Inc., Chicago, IL, USA) or MedCalc 12.3.0 (MedCalc Software, Mariakerke, Belgium). Categorical variables were compared between the groups using the χ^2^ test or Fisher's exact test, as appropriate. Differences in continuous variables were assessed using the Mann–Whitney *U*‐test. Possible confounders were explored using logistic regression analysis. The interval from AREDF diagnosis to delivery was analyzed using Kaplan–Meier survival curves, and the estimates were compared using the log‐rank test. Subgroup analysis was performed according to singleton or twin/triplet fetus, fetal sex, delivery before 26 or at or after 26 weeks, time period (1998–2006 or 2007–2015) and Doppler velocimetry results; *P* < 0.05 was considered statistically significant. The study was reported according to the STROBE guidelines^25^.

The study was approved by the Regional Research Ethics Committee at Lund University, and psychological assessment of children was performed after informed consent was given by both parents.

## RESULTS

### Perinatal outcome

**Figure 1:**
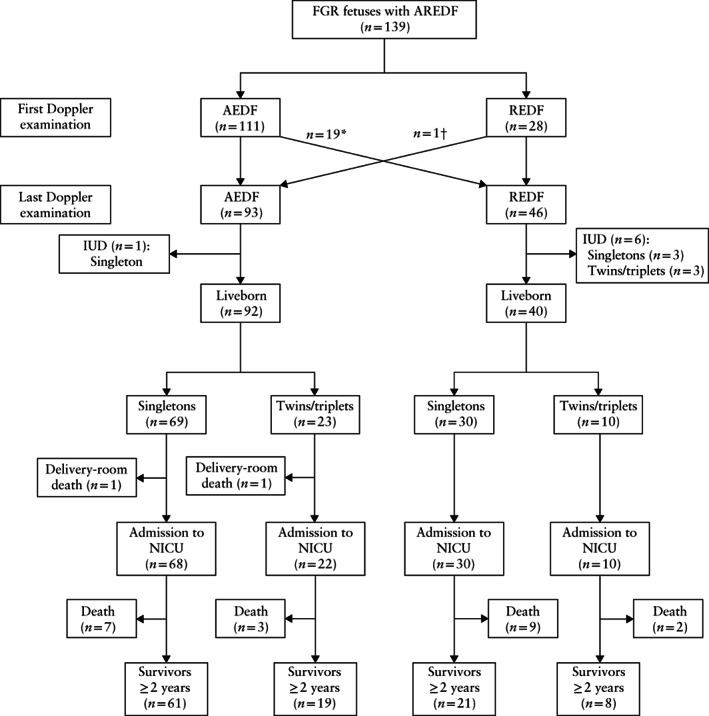
Flowchart of the study on pregnancies with early‐onset fetal growth restriction (FGR) and absent or reversed end‐diastolic flow in the umbilical artery (AREDF), delivered before 30 weeks' gestation. *Eleven singletons and eight twins. †One singleton. AEDF, absent end‐diastolic flow; IUD, intrauterine death; NICU, neonatal intensive care unit; REDF, reversed end‐diastolic flow.

During the study period, there were 139 fetuses with early‐onset FGR and AREDF in the umbilical artery (FGR group) and 946 fetuses without SGA birth weight or any known fetal Doppler changes (non‐FGR group) delivered before 30 weeks' gestation. The clinical data on the FGR and non‐FGR groups are summarized in Table 1. Maternal age, parity and the rate of gestational diabetes mellitus did not differ between the two groups. Any hypertensive disorder of pregnancy and pre‐eclampsia were more frequent in the FGR group, whereas chorioamnionitis, preterm prelabor rupture of membranes and placental abruption occurred more often in the non‐FGR group. Administration of antenatal steroids did not differ significantly between the FGR and non‐FGR groups (96% and 91%, respectively). Cesarean section was more frequent in the FGR group than in the non‐FGR group. Of the 139 FGR fetuses, 132 (95%) were liveborn and, of those, 130 (98%) were admitted to the neonatal intensive care unit (NICU) (Table 1, Figure 1). The corresponding percentages for the 946 non‐FGR fetuses were 95% and 96%. In the FGR group, seven (5.0%) fetuses were stillborn and two liveborn infants died in the delivery room, compared with 51 (5.4%) stillborn fetuses and 37 delivery‐room deaths in the non‐FGR group (difference not significant). All 132 liveborn fetuses in the FGR group were delivered by Cesarean section for fetal indication. In four cases, severe pre‐eclampsia was an additional indication for delivery. In the FGR group, six fetuses that died *in utero* were delivered vaginally. The seventh stillbirth case was in a pregnancy with growth‐restricted twin fetuses; the male fetus with AEDF developed REDF and died *in utero* at 24 + 0 weeks, and the second (female) twin had at that time positive end‐diastolic flow in the umbilical artery, but subsequently developed AEDF and was delivered by Cesarean section at 25 + 0 weeks (the latter infant is among the survivors in the FGR group).

**Table 1:** Clinical characteristics of pregnancies and infants with early‐onset fetal growth restriction (FGR) and absent or reversed end‐diastolic flow in the umbilical artery and of background population of infants without small‐for‐gestational‐age birth weight or any known fetal Doppler changes, delivered before 30 weeks' gestation

Variable	FGR (*n* = 139)	Non‐FGR (*n* = 946)	*P*
Maternal age (years)	30 (17 to 43)	31 (15 to 46)	NS
Nulliparous	77 (55)	512 (54)	NS
Twin/triplet fetus	36 (26)	267 (28)	NS
Fetal death	7 (5)	51 (5)	NS
Gestational diabetes mellitus	2 (1)	15 (2)	NS
Any hypertensive disorder of pregnancy	67 (48)	62 (7)	< 0.001
Pre‐eclampsia	57 (41)	60 (6)	< 0.001
Chorioamnionitis	0	166 (18)	< 0.001
Preterm prelabor rupture of membranes	4 (3)	314/937 (34)	< 0.001
Placental abruption	1 (1)	119 (13)	< 0.001
Antenatal steroids	124/129 (96)	746/819 (91)	NS
Gestational age at birth (days)	187 (164 to 209)	185 (154 to 209)	0.024
Gestational age at birth < 26 weeks	56 (40)	417 (44)	NS
Mode of delivery			
Cesarean section	133 (96)	551 (58)	< 0.001
Vaginal delivery	6 (4)*	395 (42)	< 0.001
Liveborn infants			
*n*	132	895	
Male sex	73/132 (55)	509/895 (57)	NS
Birth weight (g)	630 (340 to 1165)	950 (470 to 2194)	< 0.001
Birth‐weight *Z*‐score	–3.03 (–5.16 to 0.99)	0.51 (–2.0 to 4.63)	< 0.001
Placental weight (g)	240 (127 to 888)	370 (94 to 1045)	< 0.001
Non‐lethal malformation	31/132 (23)	93/895 (10)	< 0.001
5‐min Apgar score < 7	40/132 (30)	386/895 (43)	0.006
5‐min Apgar score < 4	7/132 (5)	81/895 (9)	NS
Umbilical artery pH < 7.10	2/49 (4)	20/373 (5)	NS
Umbilical artery base excess < –12 mmol/L	3/49 (6)	14/373 (4)	NS
Infants admitted to NICU			
*n*	130/132	858/895	
Need for surfactant	95/130 (73)	600/858 (70)	NS
Treatment for low arterial blood pressure	63/120 (53)	328/750 (44)	NS
Ventilation	103/130 (79)	619/858 (72)	NS
Duration of ventilation (days)	10 (1 to 47)	7 (1 to 145)	NS
Postnatal steroids	54/122 (44)	264/787 (34)	0.027
Respiratory distress syndrome	113/130 (87)	695/858 (81)	NS
Bronchopulmonary dysplasia	86/114 (75)	363/739 (49)	< 0.001
Duration of oxygen requirement (days)	76 (1 to 551)	61 (1 to 480)	0.01
Septicemia	58/128 (45)	289/845 (34)	0.017
Late‐onset septicemia	54/128 (42)	258/845 (31)	0.011
Necrotizing enterocolitis	10/130 (8)	37/855 (4)	NS
IVH/PVHI	5/128 (4)	97/842 (12)	0.008
Severe ROP (Stage ≥ 3)	10/115 (9)	85/744 (11)	NS
ROP requiring treatment	6/115 (5)	52/744 (7)	NS
Length of stay in NICU (days)	100 (1 to 312)	89 (1 to 485)	0.002

Data are presented as median (range), *n* (%) or *n*/*N* (%).

* All cases were stillborn.

IVH, intraventricular hemorrhage; NICU, neonatal intensive care unit; NS, not significant; PVHI, periventricular hemorrhagic infarction; ROP, retinopathy of prematurity.

**Figure 2:**
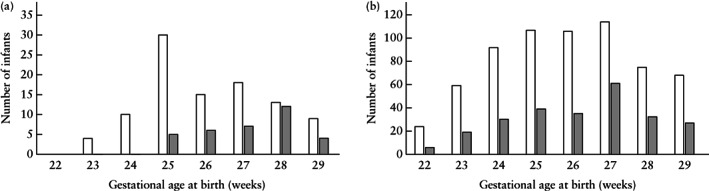
Frequency of live birth in singleton (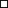) and twin/triplet (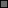) pregnancies with early‐onset fetal growth restriction and absent or reversed end‐diastolic flow in the umbilical artery (*n* = 132) (a) and pregnancies without small‐for‐gestational‐age birth weight or any known fetal Doppler changes (*n* = 895) (b), delivered before 30 weeks' gestation, according to gestational age at birth.

The follow‐up of fetuses in the FGR group according to AEDF or REDF is presented in Figure 1. The distribution of liveborn singletons and twins/triplets in the FGR and non‐FGR groups according to GA at delivery is presented in Figure 2. Of the 139 fetuses in the FGR group, 26% were twins or triplets. The corresponding percentage in the non‐FGR group was 28%. Fifty‐six (40%) and 417 (44%) infants were delivered before 26 weeks in the FGR and non‐FGR groups, respectively (difference not significant). No infants in the FGR group were delivered before 23 weeks. There was no difference in fetal sex distribution between the groups. Median GA at birth was 2 days later in the FGR group than in the non‐FGR group (187 *v*s 185 days; *P* = 0.024). Among liveborn infants, birth weight and placental weight were lower in the FGR than in the non‐FGR group (Table 1). Individual birth‐weight values according to GA are plotted in Figure S1. The frequency of 5‐min Apgar score < 7 was lower in the FGR group than in the non‐FGR group, whereas the frequency of 5‐min Apgar score < 4 and umbilical artery pH < 7.1 did not differ between the two groups. The frequency of malformations diagnosed after birth was higher in the FGR than in the non‐FGR group (Table 1); all malformations were non‐lethal and included hypospadias, atrial or ventricular septal defects and *retentio testis*.

**Figure 3:**
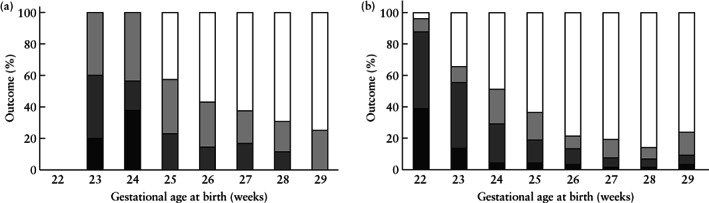
Outcome of infants with early‐onset fetal growth restriction and absent or reversed end‐diastolic flow in the umbilical artery (*n* = 139) (a) and in infants without small‐for‐gestational‐age birth weight or any known fetal Doppler changes (*n* = 946) (b), delivered before 30 weeks' gestation, according to gestational age at birth. 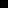, stillbirth; 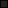, postnatal death; 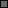, neurodevelopmental impairment (NDI); 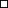, survival without NDI.

The rates of fetal death and perinatal mortality did not differ between the FGR and non‐FGR groups (5.0% *vs* 5.4% and 12% *vs* 15%, respectively) (Table 2). All seven fetal deaths in the FGR group occurred before 25 weeks (range, 23 + 5 to 24 + 6 weeks) (Figure 3). At the last examination before intrauterine demise, six fetuses had REDF and one had AEDF. In three cases, the parents decided against active management, and the remaining

four cases were twin fetuses in which expectant management was chosen in order not to compromise the health of the second twin, which had primarily normal umbilical artery blood flow.

**Table 2:** Mortality and survival in pregnancies with early‐onset fetal growth restriction (FGR) and absent or reversed end‐diastolic flow in the umbilical artery and in pregnancies without small‐for‐gestational‐age birth weight or any known fetal Doppler changes, delivered before 30 weeks' gestation

Variable	FGR (*n* = 139)	Non‐FGR (*n* = 946)	*P*
Fetal death	7 (5)	51 (5)	NS
Perinatal mortality*	17 (12)	141 (15)	NS
Overall mortality†	30 (22)	208 (22)	NS
Survival at 2 years in liveborn infants	109/132 (83)	738/895 (82)	NS
Survival at 2 years in infants admitted to NICU	109/130 (84)	738/858 (86)	NS
Survival without NDI in infants assessed at and/or after 2 years of age‡	64/104 (62)	543/656 (83)	< 0.001

Data expressed as *n* (%) or *n*/*N* (%).

* Perinatal mortality included fetal death and death within the first week postpartum.

† Overall mortality included fetal death and postnatal death up to 2 years of age.

‡ Neurodevelopmental impairment (NDI) was defined as cerebral palsy (Gross Motor Function Classification System level > 2), cognitive delay, severe hearing impairment and/or blindness.

NICU, neonatal intensive care unit; NS, not significant.

Among neonates admitted to the NICU, the rates of treatment for low arterial blood pressure, surfactant and ventilator treatment, RDS, NEC and severe ROP and the duration of ventilation did not differ between the FGR and non‐FGR groups, whereas the rates of steroid treatment, BPD and septicemia and the duration of supplemental oxygen were greater in the FGR group (Table 1). Severe IVH was significantly less prevalent in the FGR group than in the non‐FGR group (*P* = 0.008). Infants in the FGR group stayed in the NICU 11 days longer (difference between medians) than did the non‐FGR infants (Table 1).

### Long‐term outcomes

Survival at 2 years among liveborn infants and among those admitted to the NICU did not differ between the FGR and non‐FGR groups (Table 2). Overall, data on cognitive assessment were available for 760/847 (90%) children that survived to 2 years of age. Formal developmental/cognitive assessments with Bayley II or III/WPPSI‐III/WISC were performed in 404/847 (48%) children, and clinical evaluation by a neurologist/neonatologist was performed in 356/847 (42%) children. The rate of survival without NDI of assessed children after 2 years of age was lower in the FGR group than in the non‐FGR group (62% *vs* 83%; *P* < 0.001), and the differences were most pronounced among children born before 26 weeks (Figure 3). Survival without NDI correlated positively with GA at birth in both groups (correlation coefficient (*r*) of 0.97 and 0.92 in the FGR and non‐FGR groups, respectively). In the FGR group, the rate of survival without NDI increased from 0% for children born at 23–24 weeks to 75% for those born at 29 weeks. According to logistic regression analysis, in growth restricted fetuses, 5‐min Apgar score < 7 and BPD increased the risk of NDI, while increasing GA at birth and increasing birth weight decreased the risk (Table 3). In a multivariate model, after adjustment for the other variables, only low Apgar score remained a significant risk factor for NDI (odds ratio, 4.04 (95% CI, 1.49–10.96)).

**Table 3:** Association between perinatal/neonatal factors and the risk for neurodevelopmental impairment at or after 2 years of age in infants with early‐onset fetal growth restriction and absent or reversed end‐diastolic flow in the umbilical artery delivered before 30 weeks' gestation

	Odds ratio (95% CI)		
Variable	Crude	Adjusted for gestational age	Multivariate model
Gestational age at birth (in days)	0.93 (0.89–0.97)	—	0.95 (0.89–1.02)
Birth weight (in g)	0.997 (0.994–0.998)	1.0 (0.99–1.00)	1.0 (0.996–1.003)
5‐min Apgar score < 7	4.33 (1.72–10.9)	3.90 (1.46–10.41)	4.04 (1.49–10.96)
Bronchopulmonary dysplasia	3.2 (1.09–9.27)	1.55 (0.47–5.11)	1.99 (0.54–7.34)

Odds ratios were obtained from logistic regression analysis (crude, adjusted for gestational age and a multivariate model including all perinatal factors listed in the table).

Neurodevelopmental impairment was defined as cerebral palsy (Gross Motor Function Classification System level > 2), cognitive delay, severe hearing impairment and/or blindness.

After 2 years of age, the prevalence of CP did not differ between the FGR and non‐FGR groups (7% *vs* 8%) (Table 4). The rates of cognitive delay, attention deficit disorder and hearing impairment were higher in the FGR group than in the non‐FGR group. More children in the FGR than in the non‐FGR group were registered at the Pediatric Habilitation Care Service Institution. Postnatal growth failure at 2 years of age was more frequent in the FGR than in the non‐FGR group (66% *vs* 20%; *P* < 0.001) (Table 4).

**Table 4:** Neurodevelopmental and growth outcomes of survivors ≥ 2 years of age from pregnancies with early‐onset fetal growth restriction (FGR) and absent or reversed end‐diastolic flow in the umbilical artery and from pregnancies without small‐for‐gestational‐age birth weight or any known fetal Doppler changes, delivered before 30 weeks' gestation

Outcome	FGR (*n* = 109)	Non‐FGR (*n* = 738)	*P*
Cerebral palsy	8/108 (7)	55/706 (8)	NS
Cognitive delay*	35/105 (33)	72/650 (11)	< 0.001
Autism spectrum disorder	9/94 (10)	34/609 (6)	NS
Attention deficit hyperactivity disorder	19/88 (22)	51/498 (10)	0.002
Hearing impairment	9/106 (8)	26/670 (4)	0.043
Registered at Pediatric Habilitation Care Service	29/108 (27)	112/707 (16)	0.007
Growth failure at 2 years†	62/94 (66)	121/605 (20)	< 0.001

Data expressed as *n*/*N* (%).

* Cognitive delay diagnosis was based on clinical evaluation by a pediatric neurologist or neonatologist in 34% and 42% of cases in the FGR and non‐FGR groups, respectively, and on formal assessment of neurocognitive development in 62% and 46% of cases in the FGR and non‐FGR groups, respectively.

In those cases, cognitive delay was defined as Bayley II score < 70, Bayley III score < 85 or Wechsler full scale IQ < 70.

† Defined as weight > 2 SD below the mean.

### Subgroup analyses

#### *Singletons* vs *twins/triplets*

The clinical characteristics and perinatal outcomes of the singleton and twin/triplet subgroups are presented in Table S1. The median GA at delivery in the FGR twin subgroup was higher than that in the non‐FGR twin/triplet subgroup (difference of 8 days; *P* = 0.003) and the FGR singleton subgroup (difference of 10 days; *P* = 0.008). The median GA did not differ between the FGR and non‐FGR singleton subgroups. The rates of clinical perinatal variables within the singleton and twin/triplet subgroups of the FGR and non‐FGR groups were largely similar to the rates found for the respective total groups.

In the FGR group, in those born before 26 weeks, there were fewer twins/triplets than singletons (*P* = 0.018; Table S1). The median interval from Doppler diagnosis to delivery was 0 days and 2 days in the singleton and twin/triplet subgroups, respectively (*P* = 0.013). Hypertensive disorders of pregnancy (any and specifically pre‐eclampsia) were more frequent in singletons than in twins/triplets. The FGR twins/triplets had higher birth weight and placental weight than did the FGR singletons; birth‐weight deviation did not differ. The rate of late‐onset septicemia and the duration of supplemental oxygen were lower and the rate of severe IVH was higher in FGR twins/triplets than in FGR singletons.

There were no differences in mortality rates between the FGR and non‐FGR subgroups within the singleton and twin/triplet groups, or between singletons and twins/triplets within the FGR group (Table S2). The rate of survival without NDI was higher in non‐FGR singletons than in FGR singletons (*P* < 0.001); the corresponding difference between non‐FGR and FGR twins/triplets was not statistically significant. There were no differences in the mortality rate or neurodevelopmental outcome at or after 2 years of age between singletons and twins/triplets within the FGR group.

#### *Female* vs *male fetuses*

There were no significant differences in the outcome of female and male fetuses between the FGR and non‐FGR groups, with the exception of survival without NDI, which was higher in non‐FGR fetuses of both sexes (*P* = 0.002 and *P* < 0.001 for females and males, respectively; Table S2). Within the FGR group, outcome did not differ between female and male fetuses.

#### *Delivery before 26 weeks* vs *at or after 26 weeks*

The rates of fetal, perinatal and overall mortality were not significantly different between FGR and non‐FGR fetuses according to delivery before 26 weeks or at or after 26 weeks (Table S2). The rate of survival without NDI of assessed infants was higher in non‐FGR than in FGR fetuses within both GA subgroups (*P* < 0.001 for both). Within the FGR group, the rate of overall mortality was higher (*P* = 0.002) and the rate of survival without NDI was lower (*P* = 0.003) for the subgroup of fetuses born before 26 weeks than for those born at or after 26 weeks.

#### *1998–2006* vs *2007–2015*

The median GA at birth and birth weight in the FGR group did not differ when stratifying the study period into two 9‐year periods (1998–2006 and 2007–2015). The rate of surfactant administration, duration of ventilation and the rate of postnatal steroid treatment increased significantly from the first period to the second period (*P* < 0.001, *P* = 0.002 and *P* = 0.002, respectively). The rates of RDS, BPD and septicemia increased between the two periods (*P* = 0.003, *P* = 0.048 and *P* = 0.028, respectively).

The rate of perinatal mortality was lower in the FGR group (6%) than in the non‐FGR group (17%) in 1998–2006 (*P* = 0.038); during 2007–2015, it increased in the FGR group from 6% to 17% (change not significant) and did not differ significantly from that in the non‐FGR group (Table S2). The rate of survival without NDI of assessed children was significantly lower in the FGR group than in the non‐FGR group both in 1998–2006 (59% *vs* 80%; *P* = 0.002) and in 2007–2015 (64% *vs* 85%; *P* < 0.001). Within the FGR group, the differences in mortality rates and in the rate of survival without NDI between the two periods were not statistically significant.

#### Outcome according to Doppler velocimetry results

Nineteen FGR fetuses that had AEDF at entry to the study later developed REDF, and one fetus with REDF at the first Doppler examination subsequently had AEDF (Figure 1). The median GA at the first Doppler examination was 2 days earlier in the REDF than in the AEDF group (*P* = 0.01; Table S3). The interval between Doppler diagnosis of abnormal umbilical artery blood flow and delivery did not differ significantly between the AEDF and REDF groups (median of 1 day (range, 0–34 days) *vs* 0 days (range, 0–47 days)); median GA at birth also did not differ significantly between the groups. Kaplan–Meier survival curves for Doppler diagnosis to birth interval did not differ between the AEDF and REDF groups and there were no significant differences in the perinatal clinical data, with the exception of the deviation from expected birth weight, which was slightly more pronounced in the REDF group (*P* = 0.045).

The rate of survival at 2 years of liveborn infants was higher in the AEDF than in the REDF group (89% *vs* 68%; *P* = 0.006) (Table S3). The rates of CP, cognitive delay and growth failure did not differ between the two groups. There was no significant difference in the proportion of survivors without NDI among assessed infants between the AEDF and REDF groups (58% *v*s 71%).

At the last examination before birth, 31% of fetuses with REDF in the umbilical artery had absent or reversed flow during the a‐wave in the DV; the corresponding proportion in the AEDF group was 18% (difference not significant). Fetuses with a pathological DV flow pattern had significantly higher rates of overall and postnatal mortality (*P* = 0.037 and *P* = 0.022, respectively); there was no difference between the subgroups with REDF and AEDF. There was no association between NDI and DV findings. Pathological FHR tracings as a contributing indication to deliver were non‐significantly more common in the AEDF group (41%) than in the REDF group (22%). No associations between the FHR findings and mortality or NDI were found. The rates of abnormal Doppler findings in other vessels (pulsations in the umbilical vein, signs of brain sparing in the MCA, uterine artery score > 2) did not differ significantly between the REDF and AEDF groups.

## DISCUSSION

In this single‐center study, both singleton and twin/triplet fetuses with early‐onset FGR and umbilical artery AREDF were delivered before 30 weeks for fetal indication based mainly on the umbilical artery Doppler finding. Their survival rate was high (78% of all 139 fetuses; 83% of 132 liveborn fetuses) and not significantly different from that recorded in 946 non‐FGR control fetuses. The rate of survival without NDI at 2 years was 62% in the FGR group and 83% in the non‐FGR group. The proportion of children with intact survival increased with GA in both groups. Before 25 + 0 weeks, there were no survivors without sequelae in the FGR group. The outcomes did not differ between FGR singletons and twins/triplets.

The different designs of published studies on very preterm FGR fetuses with AREDF make comparison with these studies difficult. Nevertheless, on review of the literature, the outcome of growth‐restricted fetuses in the present study was comparable with or better than those in previous studies (Table S4). This is despite the fact that our study included singleton and twin/triplet fetuses with severe FGR (estimated fetal weight < 2.4^th^ percentile, AREDF and very/extremely low GA at birth).

In the European multicenter TRUFFLE study, in 503 FGR pregnancies after 26 weeks, the rate of survival was 92% and the rate of survival without impairment was 82%^26^. The outcome for the 209 fetuses with AREDF was not reported separately. In the protocol, abnormal umbilical artery Doppler findings were disregarded up to 30 weeks. Among fetuses delivered at 26–29 weeks, 6.5% of cases were stillborn and the overall mortality rate was 17%^27^. In the present study, at 26–29 weeks, there were no intrauterine deaths, the overall mortality rate was 9% and the rate of survival without NDI was 72%. The two studies cannot be compared directly because of the different designs. Still, there are some indications that our active policy based on umbilical artery Doppler could prevent fetal demise without increasing the risk of postnatal impairment.

The trial design of TRUFFLE reflects the generally accepted opinion that umbilical artery Doppler is not useful as an outcome predictor in early‐onset FGR, as it can lead to unnecessary very early interventions. Such opinion is based on published longitudinal studies on the development of various fetal parameters before delivery in very preterm FGR pregnancies^28^, ^29^, ^30^. These studies, except one^29^, investigated changes in umbilical artery pulsatility index, but did not consider the occurrence of AREDF.

Despite reports suggesting that an active approach to management of early‐onset FGR leads to higher survival rates, intervention for fetal indication before 26 weeks is not recommended^31^ and the use of umbilical artery Doppler findings as an indication for delivery before 30 weeks is discouraged^32^. The present results, supported by the experience from two smaller studies^33^, ^34^, suggest that it might be time to revise the above view and to include the occurrence of AREDF, especially the switch from AEDF to REDF, into protocols of prospective studies on the management of compromised very preterm FGR fetuses.

At birth, FGR neonates in our study were in a better immediate condition than the non‐FGR neonates, probably because all the FGR fetuses were delivered by Cesarean section. The neonatal course in the two groups was similar, with the exception of BPD and septicemia, which were more common, and severe IVH, which was less frequent, in the FGR than in the non‐FGR group. The latter finding might be associated with the higher rate of maternal pre‐eclampsia in the FGR group, as reported previously^35^. The occurrence of BPD in the FGR group was high, as also shown by others^36^. In the present study, increasing GA and birth weight decreased the risk of NDI, and BPD and 5‐min Apgar score < 7 increased the risk. This is in line with a meta‐analysis that found BPD to have a strong impact on subsequent adverse cognitive outcome^37^. However, after adjustment for GA, the association between BPD and NDI in our study was not significant (Table 3).

In accordance with previous studies^5^, ^38^, ^39^, we found that FGR fetuses with REDF had a higher rate of perinatal mortality than did those with AEDF. In contrast to all the other reports, there were no intrauterine deaths of FGR fetuses at or after 25 weeks, suggesting that the proactive obstetric management most probably prevented intrauterine death. The rates of neonatal morbidity as well as NDI at follow‐up were similar in infants with AEDF and those with REDF. Fetuses with a cofinding of an abnormal DV waveform (zero or reversed a‐wave) had an increased rate of postnatal mortality compared with those with a normal DV waveform. However, survival without NDI was not different between the two groups, which suggests that, if intrauterine death could be prevented, postnatal development would be similar irrespective of DV findings.

Our study has certain limitations. It is a retrospective observational study on the extremely rare condition of very early‐onset FGR with AREDF, with data collection covering a long period of time and possible changes in clinical practice occurring during that time. However, the rate of survival without NDI in our study did not change over time, in agreement with other reports^37^. Despite the fact that the present cohort was large, the subgroup analyses might not have had sufficient statistical power to explore the outcome variables. Postnatal development was not assessed formally in all children and was not performed at a uniform age, which is a disadvantage that we tried to circumvent by using age‐related cut‐off levels and by including information from clinical records when defining NDI. In twins/triplets, we lacked reliable information on chorionicity.

The main strength of the study is the active and rather uniform obstetric management (antenatal corticosteroids, Cesarean section) at a modern level‐3 perinatal center. Expert antenatal Doppler examinations were guaranteed, and neonatal intensive care of a very high standard was provided. A prerequisite for the proactive obstetric management of early‐onset severe FGR with AREDF is the attitude of neonatologists, who should be willing to actively treat extremely preterm infants that are more premature but less hypoxic at birth.

### Conclusions

Our findings suggest that the short‐term outcome after proactive management of very preterm fetuses with severe FGR and umbilical artery AREDF is similar to that of non‐FGR controls; however, owing to the observational study design, our findings are not definitive. The long‐term rate of NDI was higher in the FGR group than in controls, especially in infants born before 26 weeks' gestation. However, the outcome was better than that reported previously in studies on less severe FGR. The present results seem to justify the hypothesis that very preterm fetuses with umbilical artery AREDF may benefit from early intervention rather than expectant management and that umbilical artery Doppler findings could be incorporated into clinical protocols for cases very early in gestation.

## Supporting information


**Figure S1** Birth weight of neonates with early‐onset fetal growth restriction (FGR) and absent or reversed end‐diastolic flow in the umbilical artery (•) and in neonates without small‐for‐gestational‐age birth weight or any known fetal Doppler changes (o), delivered before 30 weeks in Lund during 1998–2015, according to gestational age at birth.

**Table S1** Clinical characteristics of singletons and twins/triplets in the fetal growth restriction (FGR) and non‐FGR groups

**Table S2** Mortality and survival without neurodevelopmental impairment (NDI) at or after 2 years of age in the fetal growth restriction (FGR) and non‐FGR groups, according to singleton or twin/triplet fetus, fetal sex, gestational age at delivery and time period at delivery

**Table S3** Perinatal characteristics, mortality and survival at or after 2 years of age in the FGR group, according to Doppler velocimetry in the umbilical artery

**Table S4** Reports in the literature on the outcome of very preterm growth‐restricted fetuses with absent or reversed end‐diastolic flow in the umbilical artery^3^, ^6^, ^26^, ^33^, ^34^, ^40^, ^41^, ^42^, ^43^

## Data Availability

Data available in tables and article supplementary material.
