# Supplementary figures and images for: Infant outcome after active management of early‐onset fetal growth restriction with absent or reversed umbilical artery blood flow
Source: Ultrasound Obstet Gynecol. 2021 Jun 2;57(6):931–41. doi: 10.1002/uog.23101 (PMC8252652; doi:10.1002/uog.23101)

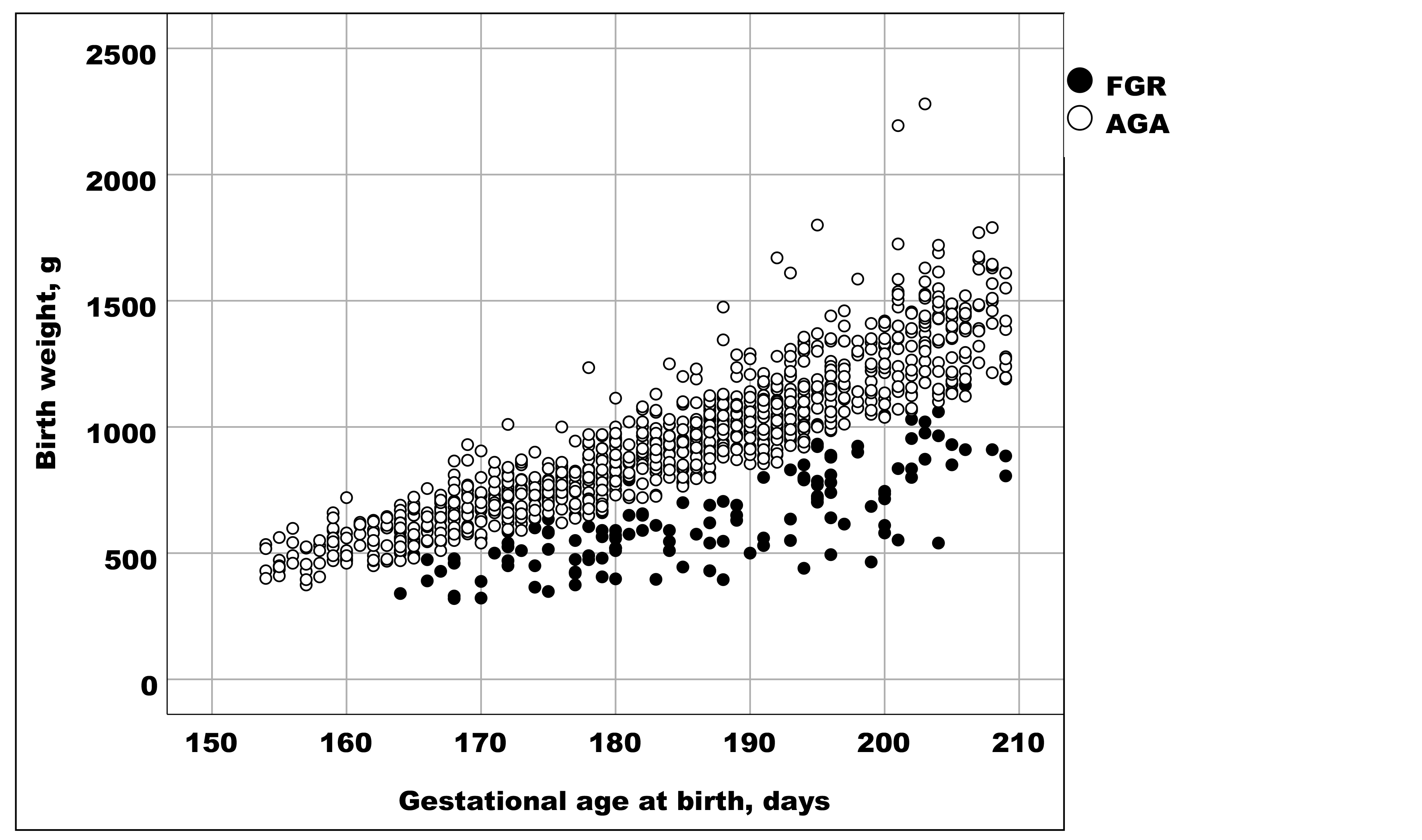

Supplement: Supplementary file 1 — Figure S1 Birth weight of neonates with early‐onset fetal growth restriction (FGR) and absent or reversed end‐diastolic flow in the umbilical artery (•) and in neonates without small‐for‐gestational‐age birth weight or any known fetal Doppler changes (o), delivered before 30 weeks in Lund during 1998–2015, according to gestational age at birth. [file UOG-57-931-s003.tif]
