# Supplementary material for: Infant outcome after active management of early‐onset fetal growth restriction with absent or reversed umbilical artery blood flow
Source: Ultrasound Obstet Gynecol. 2021 Jun 2;57(6):931–41. doi: 10.1002/uog.23101 (PMC8252652; doi:10.1002/uog.23101)
Supplement: Supplementary file 2 — Table S1 Clinical characteristics of singletons and twins/triplets in the fetal growth restriction (FGR) and non‐FGR groups [file UOG-57-931-s001.doc]

**Table S1** Clinical characteristics of singletons and twins/triplets in the FGR and non-FGR groups

|  | Singletons | | Significance of difference (p-value) | Twins / triplets | | Significance of difference (p-value) |  | Significance of difference  FGR singletons - FGR twins  (p-value) |
| --- | --- | --- | --- | --- | --- | --- | --- | --- |
|  | FGR group | Non-FGR group |  | FGR group | Non-FGR group |  |  |  |
| **N** | 103 | 679 |  | 36 | 267* |  |  |  |
| **Fetuses** |  |  |  |  |  |  |  |  |
| Gestational age at birth, days | 184  (164 – 209) | 185  (154 – 209) | ns | 194  (170 – 209) | 186  (155 – 209) | 0.003 |  | 0.008 |
| Gestational age at birth <26 GW | 48 (47) | 306 (45) | ns | 8 (22) | 111 (42) | 0.02 |  | 0.018 |
| Fetal death | 4 (4) | 34 (5) | ns | 3 (8) | 17 (6) | ns |  | ns |
|  |  |  |  |  |  |  |  |  |
| **Mothers** |  |  |  |  |  |  |  |  |
| Age, years | 30 (17 – 43) | 31 (15 – 46) | ns | 30 (23 – 41) | 30 (18 – 44) | ns |  | ns |
| Nulliparous | 58 (56) | 337 (50) | ns | 19 (53) | 175 (65) | ns |  | ns |
|  |  |  |  |  |  |  |  |  |
| **Pregnancy and delivery** |  |  |  |  |  |  |  |  |
| Gestational diabetes  mellitus | 2 (2) | 11 (2) | ns | 0 | 2 (1) | ns |  | ns |
| Any gestational  hypertension | 61 (59) | 57 (8) | <0.001 | 7 (19) | 5 (2) | <0.001 |  | <0.001 |
| Preeclampsia | 50 (49) | 55 (8) | <0.001 | 7 (19) | 5 (2) | <0.001 |  | 0.004 |
| Chorioamnionitis | 0 | 131 (19) | <0.001 | 0 | 35 (13) | 0.012 |  | ns |
| PPROM | 1 (1) | 234/676 (36) | <0.001 | 3 (9) | 80 (30) | 0.003 |  | ns |
| Placenta abruption | 1 (1) | 102 (15) | <0.001 | 0 | 17 (6) | <0.001 |  | ns |
| Antenatal steroids | 92/96 (96) | 530/584 (91) | ns | 32/33 (97) | 216/234 (92) | ns |  | ns |
| Cesarean section | 99 (96) | 390 (57) | <0.001 | 34 (94) | 161 (60) | <0.001 |  | ns |
| Vaginal delivery | 4** (4) | 289 (43) | <0.001 | 2** (6) | 106 (40) | <0.001 |  | ns |
|  |  |  |  |  |  |  |  |  |
|  |  |  |  |  |  |  |  |  |
| **Liveborn infants** |  |  |  |  |  |  |  |  |
| **N** | 99 | 645 |  | 33 | 242 |  |  |  |
| Male sex | 57 (58) | 371 (55) | ns | 16 (48) | 138 (55) | ns |  | ns |
| Birth weight, g | 587  (340 – 1165) | 944  (410 – 2185) | <0.001 | 645  (322 – 1060) | 935  (436 – 1625) | <0.001 |  | 0.021 |
| Birth weight deviation, SDS | -2.98  (-5.16 – -1.26) | -.36  (-2.0 – 4.96) | <0.001 | -3.19  (-4.81 – -0.99) | -.77  (-1.94 – 1.58) | <0.001 |  | ns |
| Placental weight | 200  (127 – 405) | 350  (94 – 924) | <0.001 | 540  (270 – 888) | 560  (200 – 1045) | ns |  | <0.001 |
| Malformation, non lethal | 22 (22) | 86 (13) | 0.031 | 10 (29) | 27 (11) | 0.006 |  | ns |
| Apgar score <7 at 5 min | 30 (30) | 274 (42) | 0.021 | 10 (29) | 112 (45) | ns |  | ns |
| Apgar score <4 at 5 min | 6 (6) | 60 (9) | ns | 1 (3) | 21 (8) | ns |  | ns |
| Umbilical artery pH | 7.28  (6.82 – 7.37) | 7.32  (6.79 – 7.51) | 0.011 | 7.29  (7.19 – 7.43) | 7.31  (6.76 – 7.46) | ns |  | ns |
| Umbilical artery base excess, mmol/L | -2.4  (-23.7 – 3.7) | -3.1  (-26.8 – 4.0) | ns | -3.1  (-15.0 – 1.6) | -3.8  (-23 – 2.0) | ns |  | ns |
|  |  |  |  |  |  |  |  |  |
| **Infants admitted to NICU** |  |  |  |  |  |  |  |  |
| **N** | 98 | 616 | ns | 32 | 242 | ns |  | ns |
| Surfactant | 72 (74) | 420 (69) | ns | 23 (72) | 180 (74) | ns |  | ns |
| Treatment for low arterial BP | 47/93 (51) | 232/541 (43) | ns | 16/27 (59) | 96/209 (46) | ns |  | ns |
| Ventilator | 80 (82) | 439 (71) | 0.026 | 23 (72) | 180 (74) | ns |  | ns |
| Ventilator, days | 12 (1 – 47) | 7 (1 – 145) | ns | 6 (1 – 31) | 6 (1 – 50) | ns |  | ns |
| Postnatal steroids | 36/94 (38) | 193/572 (34) | ns | 8/28 (29) | 71 (29) | ns |  | ns |
| RDS | 83 (85) | 491 (80) | ns | 30 (94) | 204 (84 ) | ns |  | ns |
| BPD | 65/86 (76) | 271/536 (51) | <0.001 | 21/28 (75) | 92/203 (45) | 0.006 |  | ns |
| Oxygen requirement, days | 86 (1 – 551) | 61 (1 – 480) | 0.004 | 68 (2 – 120) | 62 (1 – 341) | ns |  | 0.031 |
| Septicemia, late onset | 46/94 (49) | 183/596 (31) | 0.001 | 8/30 (27) | 74/230 (32) | ns |  | 0.036 |
| NEC | 7/94 (7) | 28/578 (5) | ns | 3/30 (10) | 9/223 (4) | ns |  | ns |
| IVH/PVHI | 1/97 /1) | 59/608 (10) | 0.002 | 4/32 (12) | 38/234 (16) | ns |  | 0.014 |
| Severe ROP, stage ≥3 | 8/86 (9) | 59/541 (11) | ns | 2/29 (7) | 26/203 (13) | ns |  | ns |
| ROP requiring treatment | 5/86 (6) | 37/541 (7) | ns | 1/29 (3) | 15/203 (7) | ns |  | ns |
| Stay in NICU, days | 102 (1 – 312) | 89 (1 – 485) | 0.001 | 98 (2 - 162) | 93 (1 – 210) | ns |  | ns |

Data are presented as median (range) or n/N (%). FGR, fetal growth restriction; AEDF, absent end-diastolic flow; REDF, reverse end-diastolic flow; PPROM, prelabor preterm rupture of membranes; GW, gestational weeks; SDS, standard deviation score (z-score); NICU, neonatal intensive care unit; BP, blood pressure; RDS, respiratory distress syndrome; BPD, bronchopulmonary dysplasia (oxygen requirement at 36 postmenstrual weeks); NEC, necrotizing enterocolitis; IVH, intraventricular hemorrhage ≥grade 3; PVHI, periventricular hemorrhagic infarction; ROP, retinopathy of prematurity. *In one case, the non-FGR twin infant was a counterpart of a twin belonging to the FGR group; **All stillbirths.
