# Supplementary material for: Infant outcome after active management of early‐onset fetal growth restriction with absent or reversed umbilical artery blood flow
Source: Ultrasound Obstet Gynecol. 2021 Jun 2;57(6):931–41. doi: 10.1002/uog.23101 (PMC8252652; doi:10.1002/uog.23101)
Supplement: Supplementary file 3 — Table S2 Mortality and survival without neurodevelopmental impairment (NDI) at or after 2 years of age in the fetal growth restriction (FGR) and non‐FGR groups, according to singleton or twin/triplet fetus, fetal sex, gestational age at delivery and time period at delivery [file UOG-57-931-s005.docx]

**Table S2** Mortality and survival without neurodevelopmental impairment (NDI) at or after 2 years of age in the FGR and non-FGR groups, according to singleton or twin/triplet fetus, fetal sex, gestational age at delivery and time period at delivery

|  | Singletons | | Significance of difference (p-value) | Twins/triplets | | Significance of difference (p-value) |  | Significance of difference FGR singletons *vs* twins/triplets |
| --- | --- | --- | --- | --- | --- | --- | --- | --- |
|  | FGR | Non-FGR |  | FGR | Non-FGR |  |  | (p-value) |
| N | 103 | 679 |  | 36 | 267 |  |  |  |
| Fetal death | 4 (4) | 34 (5) | ns | 3 (8) | 17 (6) | ns |  | ns |
| Perinatal mortality | 9 (9) | 98 (14) | ns | 8 (22) | 43 (16) | ns |  | ns |
| Overall mortality | 21 (20) | 148 (22) | ns | 9 (24) | 58 (22) | ns |  | ns |
| Survival without NDI of assessed infants | 48/80 (60) | 403/481 (84) | <0.001 | 16/24 (67) | 140/176 (80) | ns |  | ns |
|  | Females | |  | Males | |  |  | Significance of difference  FGR |
|  | FGR | Non-FGR |  | FGR | Non-FGR |  |  | females *vs* males |
| N | 66 | 407 |  | 73 | 539 |  |  |  |
| Twins/triplets  Fetal death | 19 (29)  6 (9) | 119 (29)  21 (5) | ns  ns | 17 (23)  1 (1) | 148 (27)  30 (6) | ns  ns |  | ns  ns |
| Perinatal mortality | 8 (12) | 54 (13) | ns | 9 (12) | 87 (16) | ns |  | ns |
| Overall mortality | 14 (20) | 76 (19) | ns | 16 (21) | 130 (24) | ns |  | ns |
| Survival without NDI of assessed infants | 33/49 (67) | 248/288 (86) | 0.002 | 31/55 (56) | 295/368 (80) | <0.001 |  | ns |

|  | Fetuses delivered <26 GW | |  | Fetuses delivered ≥26 GW | |  |  | Significance of difference  FGR delivered <26 *vs*  ≥26 wks |
| --- | --- | --- | --- | --- | --- | --- | --- | --- |
|  | FGR | Non-FGR |  | FGR | Non-FGR |  |  |  |
| N | 56 | 417 |  | 83 | 529 |  |  |  |
| Twins/triplets | 8 (14) | 111 (27) | ns | 28 (34) | 156 (29) | ns |  | 0.018 |
| Fetal death | 7 (12) | 41 (10) | ns | 0 | 10 (9) | ns |  | 0.001 |
| Perinatal mortality | 11 (20) | 112 (27) | ns | 6 (7) | 29 (5) | ns |  | 0.036 |
| Overall mortality | 20 (36) | 158 (38) | ns | 10 (12) | 48 (9) | ns |  | 0.002 |
| Survival without NDI of assessed infants | 14/35 (40) | 167/230 (72) | <0.001 | 50/69 (72) | 376/426 (88) | <0.001 |  | 0.003 |
|  | Period 1  1998-2006 | |  | Period 2  2007-2015 | |  |  | Significance of difference FGR delivered in period 1 *vs* period 2 |
|  | FGR | Non-FGR |  | FGR | Non-FGR |  |  |  |
| N | 62 | 422 |  | 77 | 524 |  |  |  |
| Twins/triplets | 15 (24) | 131 (31) | ns | 21 (27) | 136 (26) | ns |  | ns |
| Fetal death | 4 (6) | 31 (7) | ns | 3 (4) | 20 (4) | ns |  | ns |
| Perinatal mortality | 4 (6) | 70 (17) | 0.038 | 13 (17) | 71 (14) | ns |  | ns |
| Overall mortality | 10 (16) | 98 (23) | ns | 20 (26) | 108 (21) | ns |  | ns |
| Survival without NDI of assessed infants | 30/51 (59) | 234/292 (80) | 0.002 | 34/53 (64) | 309/364 (85) | <0.001 |  | ns |

Data expressed as n/N (%). GW, gestational weeks; AEDF, absent end-diastolic flow; REDF, reverse end-diastolic flow; NICU, neonatal intensive care unit; NDI, neurodevelopmental impairment (cerebral palsy [GMFCS >2], and/or cognitive delay, and/or severe hearing impairment, and/or blindness).
