# Supplementary material for: Infant outcome after active management of early‐onset fetal growth restriction with absent or reversed umbilical artery blood flow
Source: Ultrasound Obstet Gynecol. 2021 Jun 2;57(6):931–41. doi: 10.1002/uog.23101 (PMC8252652; doi:10.1002/uog.23101)
Supplement: Supplementary file 4 — Table S3 Perinatal characteristics, mortality and survival at or after 2 years of age in the FGR group, according to Doppler velocimetry in the umbilical artery [file UOG-57-931-s002.doc]

**Table S3** Perinatal characteristics, mortality and survival at or after 2 years of age in the FGR group, according to Doppler velocimetry in the umbilical artery

|  | AEDF | REDF | Significance of difference (p-value) |
| --- | --- | --- | --- |
| *N at inclusion to study* | 111 | 28 |  |
| Gestational age at inclusion, days | 182 (152-209) | 180 (151-203) | 0.01 |
| Interval Doppler diagnosis to delivery, days | 1 (0-34*) | 0 (0-47**) | ns |
| *N at last examination before birth* | 93 | 46 |  |
| Gestational age at last examination before birth, days | 188 (165-209) | 184 (158-204) | ns |
| Fetal death | 1 (1) | 6 (15) | 0.005 |
| Perinatal mortality | 7 (8) | 10 (22) | 0.003 |
| Overall mortality | 13 (14) | 17 (37) | 0.002 |
| *N live-born* | 92 | 40 |  |
| Gestational age at birth, days | 188 (165 – 209) | 186 (164 – 204) | ns |
| Any gestational hypertension incl. preeclampsia | 43 (47) | 24 (60) | ns |
| Twins/triplets | 23 (24) | 10 (25) | ns |
| Male sex | 50 (54) | 23 (58) | ns |
| Interval Doppler diagnosis to delivery, days | 0 (0-17) | 0 (0-47) | ns |
| Gestational age at delivery < 26 GW | 36 (39) | 20 (50) | ns |
| Cesarean section | 92 (100) | 40 (100) | ns |
| Birth weight, g | 638 (365 – 1165) | 590 (340 – 1060) | ns |
| Birth weight deviation, SDS | -2.96 (-5.16 − -1.08) | -3.58 (-5.12 − -1.26) | 0.045 |
| Malformations, non-lethal | 20 (22) | 11 (28) | ns |
| Apgar score <7 at 5 min | 28 (30) | 12 (30) | ns |
| Admitted to NICU of liveborn infants | 90 (98) | 40 (100) | ns |
| Survival ≥ 2 years | 82 (89) | 27 (68) | 0.006 |
| *N survivors at* ≥ 2 years | 82 | 27 |  |
| Cerebral palsy | 7/81 (9) | 1/27 (4) | ns |
| Cognitive delay | 26/77 (34) | 9/28 (32) | ns |
| Survival without NDI of assessed infants | 44/76 (58) | 20/28 (71) | ns |
| Weight <-2SD at 2 years | 44/68 (65) | 18/26 (69) | ns |

Data expressed as median (range) or n/N (%). AEDF, absent end-diastolic flow; REDF, reversed end-diastolic flow; NDI, neurodevelopmental impairment (cerebral palsy [GMFCS >2], and/or cognitive delay, and/or severe hearing impairment, and/or blindness). *Four fetuses with AEDF had interval to delivery 23, 25, 28, and 34 days, respectively; all developed REDF and were delivered on the day the AEDF switched to REDF. Three fetuses were twins, one of them died postnatally. **Two fetuses with REDF had interval to delivery 47 and 22 days, respectively; the first fetus was a twin delivered at 28+2 gestational weeks, the latter fetus was extremely growth restricted (birth weight SDS -5.12) delivered at 27+5 gestational weeks; the infant died postnatally.
