# Supplementary material for: Infant outcome after active management of early‐onset fetal growth restriction with absent or reversed umbilical artery blood flow
Source: Ultrasound Obstet Gynecol. 2021 Jun 2;57(6):931–41. doi: 10.1002/uog.23101 (PMC8252652; doi:10.1002/uog.23101)
Supplement: Supplementary file 5 — Table S4 Reports in the literature on the outcome of very preterm growth‐restricted fetuses with absent or reversed end‐diastolic flow in the umbilical artery 3 , 6 , 26 , 33 , 34 , 40 , 41 , 42 , 43 [file UOG-57-931-s004.docx]

**Table S4** Reports in the literature on the outcome of very preterm growth-restricted fetuses with absent or reversed end-diastolic flow in the umbilical artery

| **Reference** | **Years of study** | **FGR**  **n** | **Cesarean section** | **GA**  **at birth (weeks)** | **ARED**  **n** | **IUD** | | **Survival** | | **Survival without NDI**  **n/N (%)** | **Delivery indications** | **Comment** |
| --- | --- | --- | --- | --- | --- | --- | --- | --- | --- | --- | --- | --- |
|  |  |  |  |  |  | **of all**  **n (%)** | **of ARED**  **n (%)** | **of all**  **n/N (%)**  **(age)** | **of liveborn**  **n/N (%)**  **(age)** |  |  |  |
| **Schwarze** et al. 2005 ^40^ | 1999 - 2004 | 74 | 88% | 24-33 | 74 | 8 (11%) | 8 (11%) | 60/74 (81%)  (at discharge) | 60/66 (91%)  (at discharge) | n.a. | Maternal,  FHR,  venous Doppler | Retrospective study; single institution; ARED only |
| - subgroup <32 wks |  | 61 |  | 24-31 | 61 | 4 (7%) | 4 (7%) | 47/61 (77%) | 47/57, 82% | n.a. |  |  |
| **Hartung** et al. 2005 ^41^ | n.a.  (5-year period) | 60 | 100% of liveborn | 24-34 | 60 | 16 (27%) | 16 (27%) | 37/60 (62%)  (at discharge) | 37/44 (84%)  (at discharge) | of liveborn:  19/44 (43%)  of all:  19/60 (32% | Preeclampsia,  FHR,  abnormal DV | Retrospective study; single institution; ARED only |
| **Gerber** et al. 2006 ^33^ | 1990 - 1997 | 69 | 83% | <26-37 | 69 | 9 (13%) | 9 (13%) | 54/69 (78%) | 54/60 (90%) | of liveborn:  40/60 67%  of all:  40/69 58% | BPP, FHR, ARED | Retrospective study, single institution; follow-up at 5 years of age (1-10 y). **Policy of** **intervention on ARED;**  ARED only |
| - subgroup actively delivered |  | 35 | 100% | 26-37 | 35 | 0 | 0 | 33/35 (94%) | 33/35 (94%) | of all:  25/35 (74%) |  | Actively delivered within 24h after ARED diagnosis |
| **Baschat** et al. 2007 ^6^ | 2000 - 2006 | 604 | 97% | 24-32 | 362 | n.a. | n.a. | 456/604 (76%)  of ARED: 214/362 (59%) | 456/604 (76%)  of ARED: 214/362 (59%) | Without neonatal morbidity: 352/604 (58%) | Maternal, BPP, FHR, Doppler unspecified (31%) oligohydramnios | Prospective multicenter study, (12 centres);  **only liveborn infants** |
| - subgroup <30 wks |  | 325 | n.a. | 24-29 | 230 | n.a. | n.a. | 223/325 (69%) | 223/325 (69%) | n.a. |  |  |
| **Baschat** et al. 2009 ^3^ | 2000 - 2008 | 113 | 82% of followed-up survivors | 25-37 | 50 | 10 (9%) | n.a. | 81/113 (72%) | 81/103 (79%) | 34/72 (47%) of infants followed-up at 2 years  34/113 (30%) of all | Maternal, BPP | Prospective study, single institution; follow-up on 72 infants |
| **Story** et al. 2015 ^34^ | 2003 - 2011 | 20 | 70% of all  100% of liveborns | Med. 32 (26-39) | 15 | 6 (30%) | 3 (20%) | 12/20 (60%)  7/15 (43%)  of ARED | 12/14 (86%)  7/12 (58%)  of ARED | n.a. | ARED, FHR | Retrospective study, single institution; |
| **TRUFFLE**, **Lees** et al. 2015 ^26^ | 2005 - 2010 | 503 | 97% | Med. 30.7 (IQR  29-32) | 209 | 12 (2%) | n.a. | 463/503 (92%) | n.a. | 363/443 (82%) of all with known outcome at 2 years | Maternal,  DV changes, FHR computerized, ARED >30 wks | Prospective randomized multicenter study (20 centers);  not stratified for ARED |
| - subgroup <30 wks, Lees et al. 2013 ^26^ |  | 198 | n.a. | 26-29 | n.a. | 12 (6%) | n.a. | 154/198 (78%)  (at discharge) | 154/186 (83%)  (at discharge) | 86/186 (46%) without severe neonatal morbidity |  |  |
| **Lawin-O’Brien** et al. 2016 ^42^ | 2000 - 2015 | 245 | 40%  81% of survivors | 23 - >36  (at recruit-ment:  22-25) | 134  (77 at recruit-ment) | 122 (50%) | 57 (42%) | 101/245 (41%)  of ARED:  13/134 (10%) | 101/123 (82%)  of ARED:  13/77 (17%) | n.a. | n.a. | Retrospective multicenter study (3 centers) |
| - subgroup ≤32+0 wks |  | 181 | n.a. | 23 - ≤32+0 | n.a. | 115 (64%) | n.a. | 48/181 26% | n.a. | n.a. |  |  |
| **Dall’Asta** et al. 2020 ^43^ | 2005 - 2018 | 188 | 38%  51% (without TOP) | 23-42 | 83 | 105 (56%)  (incl. 48 TOP) | n.a. | 60/188 (32%) | 60/83 (72%) | n.a. | n.a. | Retrospective, single center, incl. anomalous fetuses.  Not stratified for ARED |
| - subgroup ≤32+0 wks  (Table S1) |  | 133 | n.a. | ≤32+0 | n.a. | 95(71%) (incl. 47 TOP) | n.a. | 25/133 (19%) | 25/38 (66%) | n.a. | n.a. |  |
| - subgroup non-anomalous |  | 136 | n.a. | Med. 28+3 (23-41) | 74 | 73 (54%)  (incl. 29 TOP) | n.a. | 48/136 (35%) | 48/63 (76%) | n.a. |  |  |
| **Morsing** et al. 2020 (**present study**) | 1998 - 2015 | 139 | 96% | 23-29 | 139 | 7 (5%) | 7 (5%) | 109/139 (78%)  (at follow-up) | 109/132 (83%)  (at follow-up) | 64/104 (62%) of assessed at ≥2 years | Primarily ARED | Retrospective, single center; **policy of intervention on ARED;**  ARED only;  26% twins |
| - subgroup <26 wks |  | 56 | 89% | 23-25 | 56 | 7 (12%) | 7 (12%) | 36/56 (64%) | 36/49 (73%) | 14/35 (40%)  of assessed  at ≥2 years |  | 14% twins |
| - subgroup singletons 26-29 wks |  | 55 | 100% | 26-29 | 55 | 0 | 0 | 50/55 (91%) | 50/55 (91%) | 46/55 (84%)  of all |  | Singletons only |

ARED, absent or reversed end-diastolic flow; BPP, biophysical profile; DV, ductus venosus; FGR, fetal growth restriction; FHR, fetal heart rate; GA, gestational age; IUD, intrauterine death; n.a., not available/not appropriate; TOP, termination of pregnancy; wks, weeks.

40. Schwarze A, Gembruch U, Krapp M, Katalinic A, Germer U, Axt-Fliedner R. Qualitative venous Doppler flow waveform analysis in preterm intrauterine growth-restricted fetuses with ARED flow in the umbilical artery – correlation with short-term outcome. *Ultrasound Obstet Gynecol* 2005; **25**: 573–579.

41. Hartung J, Kalache KD, Heyna C, Heling KS, Kuhlig M, Wauer R, Bollman R, Chaoui R. Outcome of 60 neonates who had ARED flow prenatally compared with a matched control group of appropriate-for-gestational age preterm neonates. *Ultrasound Obstet Gynecol* 2005; **25**: 566–572.

42. Lawin-O’Brien AR, Dall’Asta A, Knight C, Sankaran S, Scala C, Khalil A, Bhide A, Heggarty S, Rakow A, Pasupathy D, Papageorghiou AT, Lees CC. Short-term outcome of periviable small-for-gestational-age babies: is our counseling up to date? *Ultrasound Obstet Gynecol* 2016; **48**: 636–641.

43. Dall’Asta A, Girardelli S, Usman S, Lawin-O’Brien A, Paramasivam G, Frusca T, Lees CC. Etiology and perinatal outcome of periviable fetal growth restriction associated with structural or genetic anomaly. *Ultrasound Obstet Gynecol* 2020; **55**: 368–374.
